# Expansion of L452R-Positive SARS-CoV-2 Omicron Variant, Northern Lombardy, Italy

**DOI:** 10.3201/eid2806.220210

**Published:** 2022-06

**Authors:** Federica Novazzi, Andreina Baj, Angelo Genoni, Daniele Focosi, Fabrizio Maggi

**Affiliations:** Azienda Socio Sanitaria Territoriale, Sette Laghi, Varese, Italy (F. Novazzi, A. Baj, A. Genoni, F. Maggi);; University of Insubria, Varese (F. Novazzi, A. Baj, A. Genoni, F. Maggi);; Pisa University Hospital, Pisa, Italy (D. Focosi).

**Keywords:** COVID-19, SARS-CoV-2 Omicron variant, SARS-CoV-2, respiratory infections, viruses, Italy, coronavirus disease, zoonoses, severe acute respiratory syndrome coronavirus 2

## Abstract

We report 25 cases of infection with SARS-CoV-2 Omicron variant containing spike protein L452R mutation in northern Lombardy, Italy. Prevalence of this variant was >30% in this region, compared with <0.5% worldwide. Many laboratories are using previously developed L452R-specific PCRs to discriminate Omicron from Delta mutations, but these tests may be unreliable.

According to PANGOlin phylogeny, the SARS-CoV-2 Omicron variant of concern (VOC) consists of B.1.1.529 sublineages BA.1, in which there are 36 further sublineages; BA.2, sometimes called stealth Omicron or Omicron 2, with 3 further sublineages), and BA.3. As of April 2, 2022, among the ≈2 million Omicron sequences deposited in GISAID, spike mutation L452R (S:L452R) had been reported at <0.5% prevalence in BA.1 (425 cases), BA.1.1 (1,441), BA.1.17 (630), BA.1.15 (1,848), BA.1.1.15 (67), BA.1.15.1 (571), BA.1.14 (123), BA.1.1.1 (150), BA.1.1.2 (30), BA.1.16 (116), BA.1.1.14 (138), BA.1.1.13 (107), BA.1.1.16 (40), BA.1.1.11 (117), BA.1.13 (38), BA.1.1.12 (11), BA.1.9 (430), BA.1.12 (73), BA.1.9 (430), BA.1.12 (73), BA.1.1.10 (11), B.1.13.1 (5), BA.1.1.4 (1), BA.1.1.8 (24), BA.1.1.3 (6), BA.1.3 (3), BA.1.5 (1), BA.1.1.7 (8), BA.1.6 (4), BA.1.1.5 (1), BA.1.2 (3), BA.1.4 (1), BA.1.16.1 (1), BA.2 (125), and BA.2.3 (22).

The microbiology laboratory at Azienda Socio Sanitaria Territoriale (ASST), Sette Laghi (Territorial Social Health Authority of the Seven Lakes; Varese, Italy), which serves a wide area of the northern Lombardy region of Italy, has started a whole-genome next-generation sequencing (NGS) program for SARS-CoV-2­­–positive patients who seek care at emergency departments, as well as healthcare workers and patients in selected wards at 5 referral hospitals. During December 3, 2021–January 27, 2022, we identified 301 patients who tested positive by qualitative real-time reverse transcription PCR, then had blood samples undergo NGS; 220 samples were positive for Delta VOC and 81 for Omicron VOC. Among the Omicron cases, 25 were positive for spike mutation L452R (S: L452R) (Table). Of the sequences that we deposited in the GISAID database (https://www.gisaid.org), 17 belonged to PANGOlin sublineage BA.1, 3 to BA.1.1, 2 to BA.3, and 3 to undetermined sublineages. This proportion corresponded to a L452R prevalence of 31% (25 of 81 Omicron-positive participants in our study), compared with <0.5% worldwide. The wide heterogeneity in viral sequences excluded the likelihood of local transmission chains and supported the hypothesis of multiple introductions and convergent gene evolution.

**Table:** Patient demographics and SARS-CoV-2 phylogeny for 25 patients who tested positive for L452R-positve Omicron variant infection, northern Lombardy, Italy*

Patient no.	Date	Age, y/sex	Symptoms	NextStrain	PANGOlin	GISAID accession no.
1	2021 Dec 3	41/F	NA	21K	BA.1	EPI_ISL_9319568
2	2022 Jan 3	80/M	Fever	21K	BA.1	EPI_ISL_9306683
3	2022 Jan 3	43/F	Chest pain, diarrhea	21K	BA.1	EPI_ISL_9306774
4	2022 Jan 3	64/M	Fever and dyspnea	21K	BA.1	EPI_ISL_9319248
5	2022 Jan 3	52/F	NA	21K	BA.1	EPI_ISL_9306775
6	2022 Jan 3	55/M	Syncope (multiple sclerosis)	21K	BA.1	EPI_ISL_9306776
7	2022 Jan 3	65/F	NA	21K	BA.1	EPI_ISL_9322734
8	2022 Jan 3	48/F	Headache	21M	None	EPI_ISL_9323426
9	2022 Jan 3	31/F	NA	21M	None	EPI_ISL_9319319
10	2022 Jan 3	37/M	NA	21K	BA.1	EPI_ISL_9319384
11	2022 Jan 3	14/M	NA	21K	BA.1	EPI_ISL_9323297
12	2022 Jan 3	53/M	NA	21K	BA.1	EPI_ISL_9323497
13	2022 Jan 3	73/M	NA	21M	BA.3	EPI_ISL_9324184
14	2022 Jan 3	78/M	None	21K	BA.1	EPI_ISL_9306777
15	2022 Jan 3	82/F	None	21K	BA.1	EPI_ISL_9307474
16	2022 Jan 12	70/F	Dyspnea	21K	BA.1	EPI_ISL_9319456
17	2022 Jan 17	87/M	Right pneumonia	21K	BA.1	EPI_ISL_9324185
18	2022 Jan 19	92/M	NA	21K	BA.1	EPI_ISL_9323128
19	2022 Jan 19	2/F	NA	21K	BA.1.1	EPI_ISL_9309942
20	2022 Jan 19	47/F	NA	21K	BA.1.1	EPI_ISL_9323348
21	2022 Jan 19	45/M	NA	21M	BA.1	EPI_ISL_9324374
22	2022 Jan 19	26/M	NA	21M	None	EPI_ISL_9324445
23	2022 Jan 24	23/M	Fever (multiple sclerosis)	21K	BA.1.1	EPI_ISL_9310402
24	2022 Jan 24	22/F	NA	21K	BA.1	EPI_ISL_9323222
25	2022 Jan 24	1/F	Cough	20B	BA.3	EPI_ISL_9324518

*NA, not available.

The Omicron VOC leads to lower hospitalization and intensive care unit admission rates than some other VOCs, although it is resistant to most spike monoclonal antibodies (A. Peralta-Santos, unpub. data, https://doi.org/10.1101/2022.01.20.477163). To be effective, sotrovimab and small chemical antivirals have to be administered in the first days after onset of symptoms (D.K. Rai, unpub. data, https://www.biorxiv.org/content/10.1101/2022.01.17.476644v1), a timeframe not compatible with that needed to perform and receive results from spike or whole-genome NGS.

Attempts are underway to develop Omicron-specific PCRs, but in the interim, many laboratories are exploiting previously developed, commercially available, variant-specific PCRs to more promptly discriminate Omicron from Delta variants, an approach that relies on identifying the S: L452R mutation. Convergent evolution has led S: L452 mutations to occur in time across many different variants of interest (e.g., L452R in SARS-CoV-2 Iota and Epsilon and L452Q in Lambda), accounting for an overall 60% prevalence among SARS-CoV-2 isolates deposited in GISAID as of January 30, 2022. However, under the current simplified understanding of the variant landscape, it had been supposed that L452R mutations occurred in nearly all Delta samples across hundreds of AY sublineages, but not in Omicron samples. Our data clearly show that the approaches now being use currently are at risk of becoming unreliable for identifying variants because of gaps in knowledge.

Of note, L452R has been associated not only with resistance to some monoclonal antibodies (*1*) but also with T-cell immunity escape (N. Le Bert, unpub. data, https://doi.org/10.1101/2022.01.20.477163). The population of the northern Lombardy area has 80% coverage for 2-dose/single vaccine and 56% of residents have received an additional booster (*2*). Under such heavy selective pressure from vaccine-elicited immunity, it is not surprising that Omicron mutations leading to T-cell escape have a fitness advantage, and hence their prevalence should continue to increase.
